# Prevalence of hepatitis B and C and sensibility of a selective screening questionnaire in patients receiving chemotherapy for solid tumors

**DOI:** 10.1186/s12885-015-2033-z

**Published:** 2015-12-23

**Authors:** Mathilde Brasseur, Alexandra Heurgué-Berlot, Coralie Barbe, Cloé Brami, Jean-Baptiste Rey, Juliette Vella-Boucaud, Fadia Dabouz, Gaëtan Deslée, Florent Grange, Julien Volet, Olivier Bouché

**Affiliations:** 1 CHU Reims, Hôpital Robert Debré, Structure Interne d’Hépato-Gastro-Entérologie et Cancérologie Digestive, Avenue du Génénal Kœnig, Reims, F-51092 France; 2 CHU Reims, Hôpital Robert Debré, Unité d’Aide Méthodologique, Avenue du Génénal Kœnig, Reims, F-51092 France; 3 CHU Reims, Hôpital Robert Debré, Unité de Médecine Ambulatoire Cancérologie Hématologie, Avenue du Génénal Kœnig, Reims, F-51092 France; 4 Institut de Cancérologie Jean Godinot, Département de Pharmacie, Avenue du Génénal Kœnig, Reims, F-51100 France; 5 Université de Reims Champagne-Ardenne, Laboratoire EA4691, Avenue du Maréchal Juin, Reims, F-51100 France; 6 CHU Reims, Hôpital Maison Blanche, Maladies Respiratoires et Allergologie, Avenue du Génénal Kœnig, Reims, F-51092 France; 7 CHU Reims, Hôpital Robert Debré, Structure interne de Dermatologie, Avenue du Génénal Kœnig, Reims, F-51092 France

**Keywords:** Hepatitis C virus, hepatitis B virus, reactivation, solid tumors, HBV screening, chemotherapy

## Abstract

**Background:**

Reactivation of hepatitis B or C virus can occur in patients undergoing chemotherapy. Recommendations for selective or systematic hepatitis B virus testing prior chemotherapy for solid tumors differ. The primary aim was to determine the seroprevalence of hepatitis B or C in a low endemic country. The second objective was to assess the relevance of a questionnaire on hepatitis B/C risk factors to consider a selective screening.

**Methods:**

Patients were prospectively tested for hepatitis B/C markers. HBs antigen positive patients and isolated anti-HBc positive patients with detectable viral load received antiviral preventive treatment. Patients or physicians completed the questionnaire on infection risk factors.

**Results:**

Among the 450 patients included, 388 were tested for all serological markers and had gastrointestinal (63.7 %), lung (31.2 %) and skin (4.6 %) cancers. The prevalence of subjects exposed to hepatitis B virus was 8.5 % (33/388). One patient tested positive for HBs antigen and received preventive treatment. Prevalence of subjects exposed to hepatitis C was 1.3 % (5/388). The questionnaire sensitivity was 45.5 %, 100 % and 50 % for detecting carriers of hepatitis B, C and one or the other, respectively.

**Conclusions:**

Seroprevalence of hepatitis B was low. Selective screening with the questionnaire was insufficiently sensitive. Systematic screening with serological tests prior to chemotherapy in patients with solid tumors is therefore relevant.

**Electronic supplementary material:**

The online version of this article (doi:10.1186/s12885-015-2033-z) contains supplementary material, which is available to authorized users.

## Background

Infection with hepatitis B virus (HBV) and hepatitis C virus (HCV) is a major public health problem but many patients are not aware of their status. In France, this figure is approximately 50 % [1]. Immunosuppression induced by cancer treatment increases the risk of HBV reactivation (HBVR)[2, 3]. HCV reactivation (HCVR) is uncommon and its morbidity and mortality is less significant [4, 5].

HBVR may be asymptomatic but it can cause fulminant hepatitis and death. Additionally, HBVR may require the treatment of cancer to be modified including delaying or stopping chemotherapy [6]. This risk is present during treatment and also after stopping during the immunological rebound. The risk persists for at least 6 months after cessation [7].

The risk of HBVR depends on three main elements: host, cancer treatment and serological status [7, 8]. Effective preventive approach of HBVR is possible through antiviral treatment. While a HCV antiviral treatment is rarely compatible with chemotherapy, new findings will unquestionably result in new anti-HCV drugs.

Serological testing is the key to the prevention of HBVR. However, it can also be problematic since international recommendations differ. Hepatologists and infectious disease specialists (EASL, AASLD, APASL, CDC, NIH) recommend routine screening HBV of all candidates for immunosuppressive therapy [9–13]. These recommendations are implemented mostly by hematologists, given the frequency of HBVR associated to hematological malignancies [14, 15]. Guidelines of clinical oncology organizations (ASCO, NCCN, ESMO) suggest a selective screening in case of risk factors of hepatitis B or in patients with a strong immunosuppression (such as anti-CD20 based treatment, stem cell transplantation or lymphoma treatment) [16–18].

These differences result in inadequate screening by oncologists [14, 15] and cases of fatality. Screening before cytotoxic chemotherapy for solid tumors in countries with low prevalence of HBV is questionable and selective screening of patients at risk HBV can be assessed.

The primary endpoint of this study was to evaluate the seroprevalence of HBV and HCV in patients receiving cytotoxic chemotherapy for solid tumors. Secondary endpoints were (i) to assess the relevance of screening questions to detect risk factors of HBV and HCV and (ii) to analyze the patients with superior risk of viral reactivation.

We chose to examine these 2 objectives for HBV and HCV although HCV seems less relevant clinically.

## Methods

In a single-center cross-sectional study, all consecutive patients receiving chemotherapy for solid tumors in the Ambulatory Medicine Unit of the Reims University Hospital (France) were prospectively assessed between May 14, 2012 and July 31, 2013. Local ethics council (Reims Institutional Review Board – approval # CCTIRS 13.027 from the French *Comité Consultatif sur le Traitement de l’Information en matière de Recherche dans le Domaine de la Santé*)) according to the Declaration of Helsinki approved the study.

### Investigation scheme

Patients were informed on the objectives and methods of the study and patients provided an oral consent, accordingly with the CCTIRS approval If the patient agreed to participate, HBV and HCV serology were ordered and a screening questionnaire (Additional file 1) was submitted (self-administered or straight questionnaire, according to the patient’s level of the understanding/knowledge/intelligence) on the risk factors for exposure to HBV and/or HCV. The serology reviewed was: HBsAg, anti-HBc, anti-HBs and anti-HCV. Questions are listed in Table 1.

**Table 1:** Positive responses to risk factors questions

Risk factor questions	*n* (%)
Major surgery/bleeding prior 1992	57 (15.1)
Acupuncture, tattoo or piercing without disposable devices ^a^	55 (14.6)
Icterus	28 (7.4)
Liver disease other than cancer	23(6.1)
Transfusion prior 1992 ^b^	20 (5.3)
Relatives with viral hepatitis	18 (4.8)
Grand prematurity or serious health problem at birth	13(3.5)
Birth or medical care in countries at risk	10(2.7)
Transplantation prior 1992^a^	6 (1.6)
Blood derived product prior 1988 ^a^	5(1.3)
Hemodialysis	2(0.5)
Intravenous drug	1(0.3)
HIV+ ^a^	1(0.3)
One or more risk factor	165 (44.0)

^a^ one missing data ^b^ two missing data

**Fig. 1:**
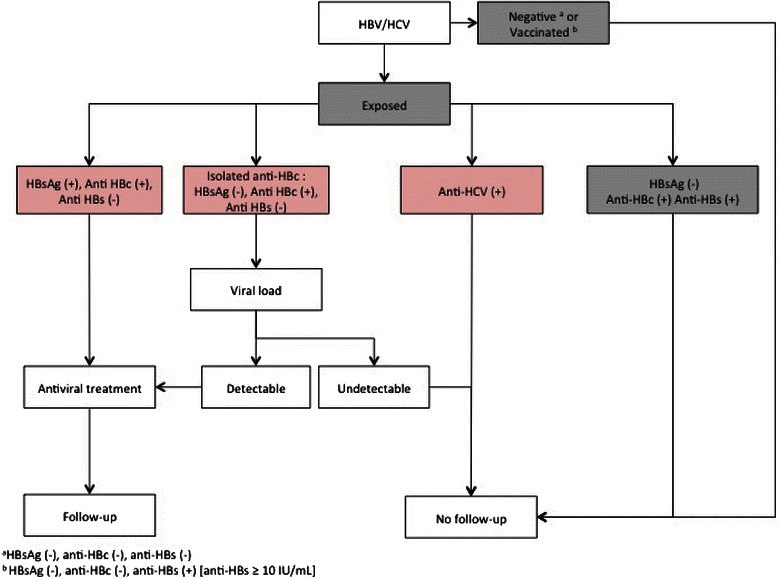
Patients’ management according to their serological status

Questionnaires were considered evaluable if (i) all responses were answered, without regard to the answer being positive or negative or (ii) incomplete with at least one positive answer to one question. Countries at risk were South East Asia, Middle East, Africa or South America. Patients’ management according to their serological status is shown in Fig. 1. HBsAg (+) patients (regardless of the viral load) and isolated anti-HBc (+) patients (with detectable viral DNA load) were considered at risk for HBVR.

### Collected data

The data collected in this study were: (i) clinical data [sex, age, tumor location, date of diagnosis, type of anti-cancer treatment (cytotoxic chemotherapy, targeted biotherapy) and therapeutic strategy (curative or palliative)], (ii) the results and dates for HBV and HCV serology, (iii) data of the screening questionnaire for risk factors and, (iv) specific clinical data of superior risk patients for reactivation (positive HBsAg, isolated anti-HBc, positive HCV or receiving anthracycline).

### Data management

All data were recorded on a standardized collection sheet specific to this study and included in a specific database (Epi Info^®^ software, v. 3.5.1, Centers for Disease Control and Prevention - CDC).

### Statistical analysis plan

Quantitative variables were described as median and range and qualitative data as number and percentage [n (%)]. Comparing patients with and without serological tests were performed using univariate analyses (t test, Wilcoxon test, KHI2 test, or Fisher exact test, as deemed appropriate). Sensitivity (Se), specificity (Sp), positive predictive value (PPV), negative predictive value (NPV) were calculated for the screening of subjects exposed to HBV, subjects exposed to HCV and subjects exposed to one or the other (each question of the questionnaire and global questionnaire). All statistical analyses were performed using SAS^®^ version 9.3 (SAS Institute Inc).

## Results

### HBV/HCV seroprevalence

Four hundred and fifty patients with solid tumors received anticancer treatment between May 14, 2012 and July 31, 2013 at the Ambulatory Medicine Unit of Reims University Hospital (France).

**Fig. 2:**
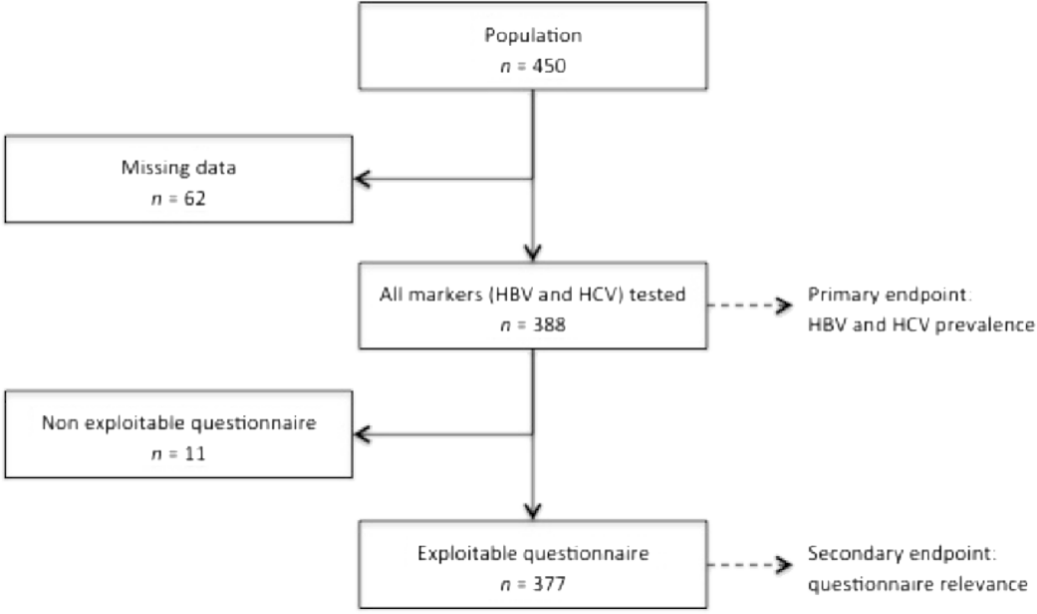
Primary and secondary endpoint populations

All HBV and HCV markers were tested in 388 of the 450 patients (86.2 %) (Fig. 2).

The characteristics of the 388 patients are presented in Table 2. More than half (63.7 %) had a gastrointestinal cancer. Among gastrointestinal cancers, half of were colorectal cancers. The most prescribed anticancer treatment was a cytotoxic doublet without biotherapy (49.7 %, *n* = 193). Most of the patients (79.9 %) were in a palliative treatment strategy.

**Table 2:** Characteristics of the patients, tumors and treatment

Characteristics	Total (*n* = 388) [*n* (%)]
Sex	
Male	261 (67.3)
Female	127 (32.7)
Age (years)	
Median (range)	64 [24–89]
Tumor localization	
Gastrointestinal	247 (63.7)
Colon rectum	124 (50.2)
Pancreas	43 (17.4)
Stomach/ cardia	34 (13.8)
Oesophagus	25 (10.1)
Biliary	15 (6.1)
Anus	4 (1.6)
Midgut	1 (0.4)
Liver	1 (0.4)
Lung	121 (31.2)
Skin	18 (4.6)
Other (Adrenocortical carcinoma)	1 (0.3)
Unknown	1 (0.3)
Treatment regimens	
Cytotoxic chemotherapy alone	288 (74.2)
Mono-chemotherapy	84 (29.2)
Bi-chemotherapy	193 (67.0)
Tri-chemotherapy	11 (3.8)
Cytotoxic chemotherapy + biotherapy	89 (22.9)
Mono-chemotherapy	11 (12.4)
Bi-chemotherapy	74 (83.1)
Tri-chemotherapy	4 (4.5)
Biotherapy alone	11 (2.8)
Therapeutic strategy	
Palliative	310 (79.9)
Curative	78 (20.1)

Missing data was due to (i) the patients’ refusal, or (ii) to deliberate or not non-prescription.

The 62 patients without HBV and HCV serology did not differ in age (*p* = 0.59), sex (*p* = 0.86), type of chemotherapy (mono or poly-chemotherapy) (*p* = 0.50) and therapeutic strategy (*p* = 0.10). Patients with gastrointestinal cancer were more likely to be tested than other patients (*p* <0.0001). Patients not tested had a significantly older diagnosis than the tested patients (*p* = 0.03).

HBV and HCV assays results are shown in Table 3. The prevalence of present or past HBV was 8.5 % (*n* = 33). Only one patient was tested positive for HBsAg. The seroprevalence of HCV was 1.3 % (*n* = 5).

**Table 3:** Hepatitis B virus and hepatitis C virus serological status

	Total (*n* = 388) [*n* (%)]
HBV exposed	33 (8.5)
Chronic HBV infection ^a^	1 (0.3)
Past HBV infection	32 (8.3)
Isolated anti-HBc ^b^	8 (2.0)
Anti-HBc (+) and	24 (6.2)
anti-HBs (+) ^c^	
HBV vaccine ^d^	56 (14.4)
HBV negative ^e^	299 (77.0)
HCV positive	5 (1.3)
HCV negative	383 (98.7)

HBV: hepatitis B virus; HCV: hepatitis C virus

^a^ HBsAg (+), anti-HBc (+), anti-HBs (−) ^b^ HBsAg (−), anti-HBc (+), anti-HBs (−) ^c^ HBsAg (−), anti-HBc (+), anti-HBs (+) ^d^ HBsAg (−), anti-HBc (−), anti-HBs (+) [anti-HBs ≥ 10 IU/mL] ^e^ HBsAg (−), anti-HBc (−), anti-HBs (−)

### Risk factors for HBV/HCV carriage

In 388 tested patients, the questionnaire was evaluable in 377 patients (83.7 % of the included cohort; *n* = 450) (Fig. 2).

Among the 11 patients without evaluable questionnaire, 9 were seronegative and 2 were vaccinated against VHB.

Positive responses for risk factors from the questionnaire are shown in Table 1. Forty-four percent of patients (165/377) had at least one risk factor.

Countries cited by the patients and considered to be at risk were the Sub-Saharan Africa (Senegal, Mauritania, Madagascar, and Guinea), the South East Asia (Indochina, Vietnam twice) and the Middle East (Iraq). This information was not available for 2 patients although they indicated a positive response to this question.

### Relevance of screening questionnaire

Se, Sp, PPV and NPV of each risk factor questions and combined (one or more risk factors) are shown in Table 4

**Table 4:** Sensibility, specificity, positive predictive value and negative predictive value of risk factors questions for hepatitis B virus positivity, hepatitis C virus positivity and hepatitis B or hepatitis C positivity

	HBV				HCV				HBV or HCV			
	Se^a^ [95 % CI]	Sp^b^ [95 % CI]	PPV^c^ [95 % CI]	NPV^d^ [95 % CI]	Se^a^ [95 % CI]	Sp^b^ [95 % CI]	PPV^c^ [95 % CI]	NPV^d^ [95 % CI]	Se^a^ [95 % CI]	Sp^b^ [95 % CI]	PPV^c^ [95 % CI]	NPV^d^ [95 % CI]
Major surgery/bleeding prior 1992	15.2	84.9	8.8	91.3	0.0	84.6	0.0	98.4	13.9	84.7	8.8	90.3
	[2.9-27.4]	[81.1-88.7]	[1.4-16.1]	[88.2-94.3]		[81.0-88.3]		[97.1-99.8]	[2.6-25.2]	[80.9-88.5]	[1.4-16.1]	[87.0-93.5]
Acupuncture, tattoo or piercing without disposable devices	12.5	85.2	7.3	91.3	40.0	85.7	3.6	99.1	14.3	85.3	9.1	90.6
	[1.0-24.0]	[81.4-88.9]	[0.4-14.1]	[88.2-94.4]	[0.0-82.9]	[82.1-89.2]	[0.0-8.6]	[98.0-100]	[2.7-25.9]	[81.5-89.1]	[1.5-16.7]	[87.4-93.8]
Icterus	12.1	93.0	14.3	91.7	40.0	93.0	7.1	99.1	13.9	93.2	17.9	91.1
	[1.0-23.3]	[90.3-95.7]	[1.3-27.2]	[88.8 -94.6]	[0.0-82.9]	[90.4-95.6]	[0.0-16.7]	[98.2-100.0]	[2.6-25.2]	[90.6-95.9]	[3.7-32.0]	[88.1-94.1]
Liver disease other than cancer	18.2	95.1	26.1	92.7	60.0	94.6	13.0	94.9	22.2	95.6	34.8	92.1
	[5.0-31.3]	[92.8-97.3]	[8.1-44.0]	[89.6 -95.1]	[17.1-100.0]	[92.3-96.9]	[0.0-26.8]	[92.6-97.1]	[8.6-35.8]	[93.4-97.8]	[15.3-54.2]	[89.2-94.9]
Transfusion prior 1992	3.0	94.4	5.0	91.0	0.0	94.6	0.0	98.6	2.8	94.4	5.0	90.1
	[0.0-8.9]	[92.0-96.9]	[0.0-14.6]	[88.0-94.0]		[92.3-96.9]		[97.4-99.8]	[0.0-8.1]	[91.9-96.8]	[4.6-14.6]	[87.0-93.2]
Relatives with viral hepatitis	6.1	95.3	11.1	91.4	0.0	95.1	0.0	98.6	5.6	95.3	11.1	90.5
	[0.0-14.2]	[93.1-97.6]	[0.0-25.6]	[88.5 -94.3]		[93.0-97.3]		[97.4-99.8]	[0.0-13.0]	[93.0-97.5]	[0.0-25.6]	[87.5-93.5]
Grand prematurity or serious health problem at birth	0.0	96.2	0.0	90.9	0.0	96.5	0.0	98.6	0.0	96.2	0.0	97.6
		[94.2-98.2]		[88.0-93.9]		[94.6-98.4]		[97.4-99.8]		[94.1-98.2]		[95.9-99.2]
Birth or medical care in countries at risk	3.0	97.4	10	91.3	0.0	97.3	0.0	98.9	2.8	97.4	10	90.4
	[0.0-8.9]	[95.7-99.1]	[0.0-28.6]	[88.4-94.2]		[95.7-99.0]		[97.4-99.8]	[0.0-8.1]	[95.6-99.1]	[0.0-28.6]	[87.4-93.5]
Transplantation prior 1992	0.0	98.3	0.0	91.1	0.0	98.4	0.0	98.6	0.0	98.2	0.0	90.2
		[96.9-99.6]		[88.2-94.0]		[97.1-99.7]		[97.5-99.8]		[96.8-99.6]		[87.2-93.3]
Blood derived product prior 1988	3.0	98.8	20.0	91.4	20	98.9	20	98.9	2.9	98.8	2	90.5
	[0.0-8.9]	[97.7-100.0]	[0.0-55.1]	[88.5-94.2]	[0.0-55.1]	[97.9-100]	[0.0-55.1]	[97.9-100.0]	[0.0-8.4]	[97.7-100]	[0.0-55.1]	[87.6-93.5]
Hemodialysis	0.0	99.4	0.0	91.2	0.0	99.5	0.0	98.7	0.0	99.4	0.0	90.4
		[98.6-100.0]		[88.3 -94.1]		[98.7-100.0]		[97.5-99.8]		[98.6-100.0]		[87.4-93.4]
Intravenous drug	3.0	100.0	100.0	91.5	0.0	99.7	0.0	98.7	2.8	100.0	100.0	90.7
	[0.0-8.9]			[88.7 -94.3]		[99.2-100.0]		[97.5-99.8]	[0.0-8.1]			[87.7-93.6]
Human Immunodeficiency Virus +	0.0	99.7	0.0	91.2	0.0	99.7	0.0	98.7	0.0	99.7	0.0	90.4
		[97.1-100.0]		[88.3-94.1]		[99.2-100.0]		[97.5-99.8]		[99.1-100.0]		[87.4-93.4]
One or more risk factor	45.5	56.4	9.1	91.5	100.0	56.9	3.0	100.0	50.0	56.8	10.9	91.5
	[28.5-62.4]	[51.2-61.6]	[4.7-13.5]	[87.8-95.3]		[51.8-61.9]	[0.4-5.6]		[33.7-66.3]	[51.5-62.0]	[6.2-15.7]	[87.7-95.2]

^a^ sensibility; ^b^ sensitivity; ^c^ positive predictive value; ^d^ negative predictive value

.

The most sensitive item was history of liver disease (22.2 % [8.6-35.8]). The two most specific items were HIV seropositivity (100 %) and intravenous drug use (99.7 % [99.1-100.0], limited to thèse two patients).

### Patients with superior risk of reactivation

The only patient tested HBsAg positive was treated with pemetrexed for advanced non-small cell lung carcinoma diagnosed 10 months before performing serology. This patient was unaware of his status. He did not present with any risk factor for HBV carriage. DNA viral load was undetectable but, in accordance with recommendations, the patient was treated with entecavir (0.5 mg per day). Regular monitoring was conducted with a hepatologist. HCV serology was negative.

A therapeutic break was proposed due to prolonged tumoral stability. Entecavir was stopped 6 months after cessation of chemotherapy while monitoring DNA viral load continued for 3 months. No viral reactivation was demonstrated at 20 months of follow-up after initiation of the treatment.

Eight patients had isolated anti-HBc. The DNA viral load achieved in five patients was negative; the other 3 patients declined rapidly and expired from their cancer. One of these 8 patients had a risk factor for HBV carriage (born in Guinea); assessment of the DNA viral load could not be performed in this patient. No patient was seropositive for HCV. In accordance with the guidelines, patients having a negative DNA viral load did not receive antiviral treatment.

Five patients were anti-HCV (+). This status was known by two of the five patients and were followed untreated. The three who were unaware of their status had identified a risk factor in the questionnaire (tattoo, albumin prior to 1988 and liver disease history).

Only 4 patients received anthracylines. Three patients received epirubicin 50 mg/m^2^ combined with oxaliplatin and capecitabine (EOX regimen) for metastatic gastric cancer. Two were negative for HBV, one being properly vaccinated, and were seronegative for HCV. No indication of risk factors for HCV or HBV carriage were noted for these patients. A patient received doxorubicin 50 mg/m^2^ (day 1 and day 22) combined with streptozotocin for metastatic pancreatic neuroendocrine tumor (Zollinger Ellison Syndrome). The patient refused serological assays and participation in the questionnaire. No significant cytolysis was seen retrospectively during the 16 months following the initiation of polychemotherapy.

## Discussion

### HBV/HCV seroprevalence

This study presents a prospective assessment for the seroprevalence of HBV and HCV in patients receiving cytotoxic chemotherapy for solid tumors in a country with low endemicity of HBV and HCV. Other studies examining these factors exist but the countries assessed were high endemic [3, 6, 8] or intermediate endemic [19–21] prevalence.

In our population, the prevalence of chronic HBV [HBsAg (+)] was lower than that of the general population in France (0.3 % vs. 0.65 %) [1] but higher (0.3 % vs. 0.11 %) than the French hospital prevalence [22]. The target population for selective screening consists of subjects exposed to HBV. Seroprevalence in these patients (8.5 %) was identical to the French general population favoring the extrapolation of these results [1].

The seroprevalence of HCV (1.3 %) was higher when compared to both the French general (0.84 %) [1] and hospital populations (0.33 %) [22]. One contributing factor for this observation may have been due to an increased risk of receiving a transfusion or other procedural contamination prior to 1992 due to the age of these patients.

### Rationale for selective screening

Systematic HBV screening in patients with solid tumors may not be as cost-effective as chemotherapy when the HBVR and the prevalence of HBV are low [23]. The estimated cost of care and cost-effectiveness ratio is considered relevant. Cost effectiveness depends on the care system of the country and therefore studies of cost -effectiveness are difficult to extrapolate from one country to another.

The HBVR risk depends on three components: the type of solid tumor, the treatment and the serological status. This risk was more prevalent in breast cancer (estimated up to 41 %) [6, 7] and hepatocellular carcinoma patients (36 %) [24]. In other solid tumor, HBVR risk was approximately 16 % in prospective studies [3, 7]. Our population did not include patients with breast cancer and included only one patient with liver cancer; making the RVHB risk low.

The highest rates of HBVR in patients undergoing chemotherapy for solid tumors occurred with anthracycline-based regimen [6, 7]. This type of regimen is rarely used outside of breast cancer (FEC/AC); as reflected in our study with only 4 patients who received an anthracycline.

According to the serological status, 9 patients were considered at risk of reactivation (2 %), which shows a possible benefit of screening. But the only patient treated was HBsAg (+). Among 388 screened patients only one was treated, which probably decreases the relative cost-effectiveness of a routine screening. Moreover, the only solid tumor cost-efficiency study suggested a screening with HBsAg alone [23], since the HBsAg (+) patients are most at risk of HBVR.

The screening questionnaire shows the need for oncologists to be educated on risk factors for carriage of HBV/HCV since only 33 % identified the place of birth in endemic areas as a risk factor (while being the main risk [15, 25]).

We found that our population was ideal for a selective screening. To our knowledge, and from the time of this publication, there have been no published evaluations for the selective screening process considering sensitivity, specificity, and predictive value in a low endemic country for viral hepatitis.

### Relevance of targeted screening on HBV/HCV risk factors

Selective screening is performed in two steps: a pre-screening (questionnaire), followed by the serological test. A quality pre-screening test will focus on sensitivity while a quality serological test will focus on specificity.

In the present study, the sensitivity for HBV was 45.5 %, leaving out more than half of seropositive HBV patients. Additionally, the only patient HBsAg (+) would not have been identified and treated using a selective strategy since he had no risk factor on the questionnaire. The overall sensitivity (exposed to HBV or HCV) was 50 %, therefore insufficient. Sensitivity was 100 % for HCV but, as stated previously, HCV infection is not the main concern. The specificity of the questionnaire was also insufficient (56 %, approximately).

The probability of being seropositive for HBV, HCV and one or the other in case of positive questionnaire was very low since the overall PPV did not exceed 11 %. Recalling the differences in clinical outcome of RHCV, the screening recommendations for HBV and HCV cannot be the same.

Few studies have assessed the relevance of targeted screening on HBV / HCV risk factors [26–28]. One study with pregnant women in the United States was designed to test a questionnaire recommended by the ACIP (Advisory Committee on Immunization Practices). This questionnaire was used for 692 parturient women among whom 8.5 % were HBV positive. The sensitivity was less than 60 % for screening carriers of HBV[28] . These findings led to the current policy of universal prenatal screening.

### Limitations

#### This study is missing data

It was noted that serologic test were not ordered due to either (i) deliberate from relevance deemed insufficient by the oncologist because of the entry into advanced palliative/terminal phase or (ii) unintentional due to lack of awareness of oncologists despite the study implementation. Without a doubt, there is an underestimation for the risk of RHBV by oncologists [15] although it is possible to conclude that digestive oncologists may be more informed on this since patients with gastrointestinal tumors were more likely to be tested in our study.

This questionnaire is lacking in sensitivity. First, the main mode (one third of cases) of transmission of HBV,[1] is sex. Except for HIV seropositivity and *noting relatives infected with viral hepatitis, sexually transmitted infections* were intentionally excluded from the questionnaire due to the subjectivity of the concept of unsafe sex and the lack of reliability of predictable responses. Secondly, lack of sensitivity of our survey for HBV could be expected; actually, in one third of cases the mode of transmission of hepatitis B is not found [1]. However, it had to be proven; we can conclude that, in our population, if a screening must be done, it has to be systematic, especially since serologic test is sensitive, specific and inexpensive.

The CDC (Centers for Disease Control and Prevention) developed a on-line questionnaire specifically for these risk factors which was completed by patients [29]. The estimated time to complete this questionnaire was 5 minutes [29]. This questionnaire is being used in an ongoing prospective study at the MD Anderson Cancer Center (USA), which is to include 3,400 patients prior to chemotherapy and to compare both strategies in terms of sensitivity, specificity and cost-effectiveness [30]. This questionnaire could have a different sensitivity. The reliability of patients’ anamnestic data and their lack of education for risk of contamination (i.e. infected patients who do not recognize or report on risk factors) remain to be an issue.

## Conclusions

In conclusion, two main observations can be drawn from this study: (i) a very low prevalence of chronic HBV in our population with only one patient who received antiviral treatment and (ii) a lack of sensitivity of the screening questionnaire on viral hepatitis risk factors. These results support the relevance of routine screening with serological tests prior chemotherapy in patients with solid tumors.

Another type of selective screening based on risk of viral reactivation (relying on the type of chemotherapy and cancer) is possible; more robust data are needed to determine the incidence and predictive factors for HBVR. Development of a register to track viral reactivation cases would be useful.

Expecting consensual recommendations that will harmonize and simplify practices, oncologists’ education and collaboration with hepatologists must be initiated or continued.

## Additional file

Additional file 1: **QUESTIONNAIRE.** (PDF 54.5 kb)

## Abbreviations

HBV: hepatitis B virus
HCV: hepatitis C virus
HBVR: hepatitis B virus reactivation
HCVR: hepatitis C virus reactivation
EASL: European Association for the Study of the Liver
AASLD: American Association for the Study of Liver Diseases
APASL: The Asian Pacific Association for the Study of the Liver
CDC: Centers for Disease Control and Prevention
NIH: National Institutes of Health
ASCO: American Society of Clinical Oncology
NCCN: The National Comprehensive Cancer Network
ESMO: European Society for Medical Oncology
