# Supplementary material for: Prevalence of hepatitis B and C and sensibility of a selective screening questionnaire in patients receiving chemotherapy for solid tumors
Source: BMC Cancer. 2015 Dec 23;15:999. doi: 10.1186/s12885-015-2033-z (PMC4688993; doi:10.1186/s12885-015-2033-z)
Supplement: Additional file 1: — QUESTIONNAIRE. (PDF 54.5 kb) [file 12885_2015_2033_MOESM1_ESM.pdf]

QUESTIONNAIRE
(translated from French)

Last name (first 3 letters): |__|__|__|

First name (first 3 letters): |__|__|__|

Birth date: |__|__| |__|__| 19|__|__|

Are you or have you been involved in the following situations?

1. Transfusion (red cells, platelets or plasma) before 1992? ☐ NO ☐ YES

2. Graft (tissue, organ or cells) before 1992? ☐ NO ☐ YES

3. Major surgery, major bleeding, obstructed labor (traumatic), ICU stay before 1992? ☐ NO ☐ YES

4. Serious health problem at birth or premature birth? ☐ NO ☐ YES

5. Infusion of albumin, immunoglobulins, clotting factors before 1988? ☐ NO ☐ YES

6. Liver disease (other than cancer)? ☐ NO ☐ YES
   iI yes which one: ………………………………………………

7. « Jaundice »? ☐ NO ☐ YES

8. Someone you know suffering from viral hepatitis? ☐ NO ☐ YES

9. Hemodialysis? ☐ NO ☐ YES

10. Intravenous drug use? ☐ NO ☐ YES

11. HIV seropositivity? ☐ NO ☐ YES

12. Tattoo, piercing, acupuncture without disposable devices? ☐ NO ☐ YES

13. Birth or medical care in Southeast Asia, Middle East, Africa or South America? ☐ NO ☐ YES
    If yes, specify country: ………………………………………….
